# Analyzing Healthcare Processes with Incremental Process Discovery: Practical Insights from a Real-World Application

**DOI:** 10.1007/s41666-024-00165-6

**Published:** 2024-06-24

**Authors:** Daniel Schuster, Elisabetta Benevento, Davide Aloini, Wil M. P. van der Aalst

**Affiliations:** 1 Data Science & Artificial Intelligence, Fraunhofer Institute for Applied Information Technology FIT, Schloss Birlinghoven, 53757 Sankt Augustin, Germany; 2 Chair of Process and Data Science, RWTH Aachen University, Ahornstraße 55, 52074 Aachen, Germany; 3 Department of Energy, Systems, Territory, and Construction Engineering, University of Pisa, Largo Lucio Lazzarino, Pisa, 56122 Italy; 4 Centro di Servizi Polo Universitario “Sistemi Logistici”, University of Pisa, Via dei Pensieri 60, Livorno, 57128 Italy

**Keywords:** Process mining, Business process management, Interactive process discovery, Domain knowledge, Process modeling

## Abstract

**Abstract:**

Most process mining techniques are primarily automated, meaning that process analysts input information and receive output. As a result, process mining techniques function like black boxes with limited interaction options for analysts, such as simple sliders for filtering infrequent behavior. Recent research tries to break these black boxes by allowing process analysts to provide domain knowledge and guidance to process mining techniques, i.e., hybrid intelligence. Especially, in process discovery—a critical type of process mining—interactive approaches emerged. However, little research has investigated the practical application of such interactive approaches. This paper presents a case study focusing on using incremental and interactive process discovery techniques in the healthcare domain. Though healthcare presents unique challenges, such as high process execution variability and poor data quality, our case study demonstrates that an interactive process mining approach can effectively address these challenges.

**Graphical Abstract:**

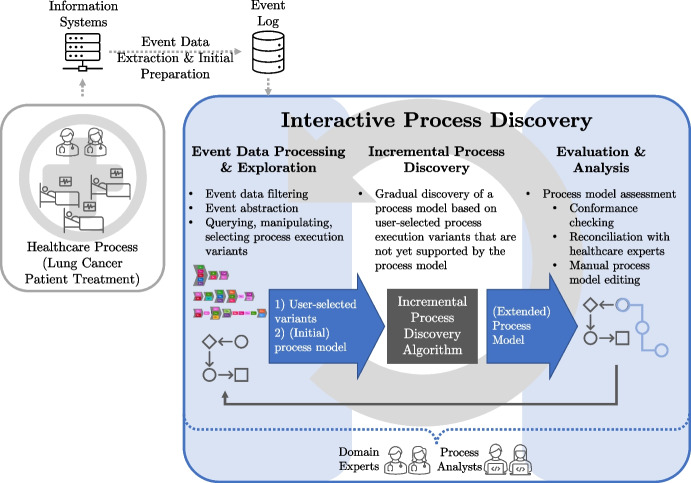

## Highlights

- A case study on the application of novel incremental/interactive process discovery techniques to analyze the treatment process of lung cancer patients
- A novel technique to integrate domain knowledge in the form of ordering constraints into concurrency-aware process execution variants
- Practical implications, open challenges, and lessons learned for analyzing knowledge-intensive processes with interactive/incremental process discovery techniques

## Introduction

Healthcare organizations need to improve care quality and effectiveness while managing costs [1]. Streamlining operational processes reduces waiting times, minimizes delays, optimizes resource allocations, and provides timely care for better patient outcomes. In short, process improvements in healthcare organizations are of great importance, as shown by studies [2–4].

The ongoing digitalization in the healthcare sector allows organizations to analyze their processes in a data-driven fashion [5, 6]. Technologies, such as electronic health records [7], blockchain [8], IoT [9], eHealth [10], and telemedicine [11], are increasingly being integrated into healthcare processes, enabling organizations to collect an ever-increasing volume and variety of patient and process data  [12]. The growing availability of such data opens up new opportunities for analyzing healthcare processes through data-driven approaches, i.e., *process mining* [1, 13]. Process mining provides valuable insights for healthcare organizations regarding their processes to eventually optimize them regarding performance, anomalies, and resource allocations [14].

Despite the significant potential of process mining for analyzing healthcare processes [14], the unique characteristics of the healthcare sector and its processes impose specific challenges that require attention when applying process mining [15, 16]. Munoz et al. [14] emphasize two significant challenges: (1) high variability of individual process executions of the same process and (2) poor quality of data generated by hospital information systems. First, healthcare processes are inherently complex, i.e., *knowledge-intensive* [17]. Further, they exhibit significant variability due to variations in patient characteristics, response to treatments, and the experience of physicians and other healthcare professionals involved  [15]. Consequently, almost all patient cases are unique, and several possible treatment pathways exist for a given medical condition [14]. Second, data quality is a significant concern in the healthcare sector. Studies have found that healthcare data sets frequently have missing or incorrect timestamps or events, often caused by data entry or collection errors [1, 14, 18–20]. These issues can arise due to excessive workload, inadequate training, and extensive manual recording of performed activites [21–23]. These two challenges, i.e., high variability of individual process executions and poor data quality with regard to temporal information, can negatively impact the reliability and effectiveness of process mining techniques in the healthcare sector if not appropriately managed [14].

The inclusion of domain experts in process mining analysis is an expansive issue that has been progressively captivating the interest of researchers [22, 24]. This paper focuses on incorporating domain experts and their knowledge in process discovery—a key type of process mining. Traditional automated process mining techniques [25–27], predominant in industry and academia, are poorly prepared to handle low-quality event logs from highly variable processes. In addition, these approaches do not provide ways to leverage domain knowledge or use it interactively. Indeed, these approaches often produce so-called spaghetti models [13] that are incomprehensible, challenging to interpret for healthcare managers, and of little informative value. Therefore, new *interactive process discovery* approaches emerged, which allow the incorporation of domain knowledge into the discovery of process models [22, 24, 28]. Combining domain knowledge from process stakeholders and event data, i.e., hybrid intelligence, can improve the results’ quality and enable better process modeling [29]. In the healthcare context, where physicians possess profound domain knowledge, integrating this knowledge within the process discovery phase can provide critical advances compared to traditional automated discovery techniques  [25, 26]. However, the development and use of these interactive approaches are still limited, and their suitability in supporting the discovery of healthcare processes is mainly unexplored.^1^

Given the limited evidence on the effectiveness and suitability of interactive process mining techniques, especially incremental process discovery, further investigation is required. This case study contributes to the literature by analyzing a real-world healthcare process, i.e., the treatment of lung cancer patients, using an interactive open-source process discovery tool and identifying best practices and challenges based on it. Further, this study addresses state-of-the-art process mining and business process management challenges. In [30], the authors list various central problems in business process management. One of the challenges identified is determining a suitable detail level for process activities. To overcome this, we utilize the domain knowledge of physicians, establish hierarchies of activities, and carefully choose the level of abstraction in consultation with the doctors and analysis objectives. Through this and our general approach, we address another problem: “augmenting process mining with common sense and expertise” [30].

**Fig. 1:**
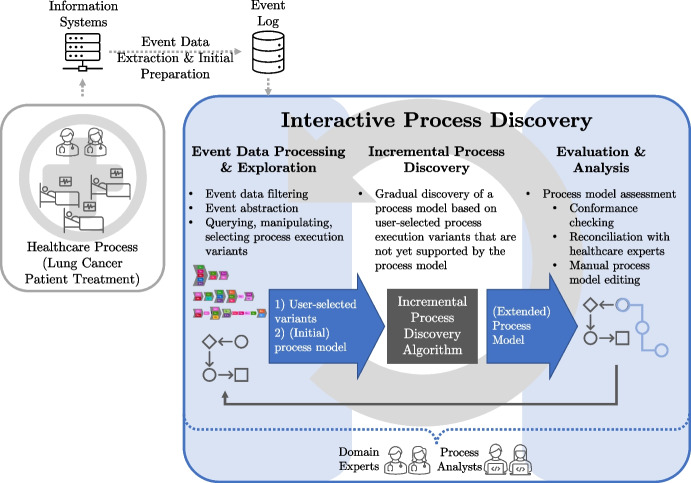
Overview of the case study conducted. Starting from an event protocol describing the treatments of lung cancer patients, we interactively and incrementally discover a process model that summarizes the complex and highly individual sequence of the different treatment steps

This study investigates the use of *incremental process discovery* for the analysis of knowledge-intensive healthcare processes. To achieve this, we conducted a study that analyzes a real event log documenting the treatment of lung cancer patients. Figure 1 outlines the general approach of this study. Besides event data extraction and initial preparation, the primary approach is represented by the outlined blue box, wherein a process model is gradually discovered from the available event data. In each cycle, domain experts or process analysts selectively choose individual process execution variants to be added to the evolving process model. Through this gradual approach, the process model is incrementally discovered so that users can better understand and comprehend how process models are learned from data.

To analyze the event data, we utilize the *open-source process mining tool Cortado* [31] for the whole central part of the study; see the blue outlined box in Fig. 1. Combining interactive data preparation techniques with *incremental process discovery* [32], Cortado offers comprehensive features to address healthcare issues. In addition to the real study, we propose a novel technique for integrating domain knowledge into process execution variants essential for interactive process discovery. We created this technique within the scope of the study, and it is considered an additional contribution. Nevertheless, the proposed technique can be universally applied also in other domains when analyzing event data. Therefore, we implemented the proposed technique in Cortado [31] In short, this study is one of the first to use such an innovative interactive process discovery approach in a complex healthcare setting. Incorporating domain knowledge can mitigate the variability and complexity inherent in knowledge-intensive and unstructured processes.

The remainder of this paper is structured as follows. Section 2 presents related work, while Sect. 3 introduces general terms and concepts. In Sect. 4, we provide an overview of the Cortado tool and its functionality. Section 5 presents the research design of the conducted case study, while Sec. 6 presents the corresponding results. In Sect. 7, we discuss the study’s results and derive practical implications. Finally, Sect. 8 concludes this paper.

## Related Work

In recent years, process mining has been widely used in healthcare, as evidenced by several literature reviews on the topic  [14, 33–35]. These applications of process mining within the healthcare domain include identifying correct patient flows  [3], analyzing process performance  [4], evaluating adherence to clinical guidelines  [36], and predicting patient outcomes based on process execution  [37]. In the following, we focus on interactive process mining techniques.

Healthcare literature has recently explored interactive techniques that utilize event log data and domain expertise [22]. However, despite the potential advantages, this research is still in its early stages. To the best of our knowledge, only a few examples have proven the practicality and benefits of interactive methods in a complex real-world scenario like healthcare.

Martin et al. [38] propose an interactive data cleaning approach consisting of three steps, i.e., data-based data quality assessment, discovery-based data quality assessment, and data cleaning heuristics. The authors tested their approach with a case study of an outpatient clinic’s appointment system. In this approach, users can guide the cleaning of the event log by exploiting their domain knowledge. However, the focus of the approach is only on data cleaning. In addition, the proposed approach requires an analyst with sufficient experience in the control of interactive data cleaning.

On the contrary, only two approaches focus on the interactive discovery of the process model. In  [19], a methodology for using process mining technologies over an interactive pattern recognition framework for supporting the iterative design of clinical pathways [39] for chronic diseases is presented. However, while the experts cannot be directly involved in discovering the process model, they can still iteratively use the developed approach to visualize it and selectively make informed decisions. In [21], the authors demonstrate the effectiveness of the interactive process discovery tool developed by [29] in modeling real-life healthcare processes from noisy event logs. This approach allows the experts to actively discover the process model using their domain knowledge and the information recorded in the event log. Although the interactive tool is practical, it only allows one activity to be added at a time during the construction of the model. In addition, it requires prior knowledge of Petri nets as a modeling formalism and thus can take longer to learn. The critical difference to incremental process discovery is that in [29], individual activities are interactively placed in a process model considered under construction. In contrast, incremental process discovery, subject to his case study, gradually adds user-selected process execution variants, which consist of various activities, to a model under construction. From the user’s point of view, there is a big difference between dealing with individual activities gradually and dealing with execution variants that describe complete process executions from start to end. As pointed out in [40], domain experts often think of single, specific process executions. Indeed, when considering process execution variants, the analysis shifts towards a broader view. Instead of examining individual activities in isolation, the emphasis is on capturing complete process executions. Therefore, the incremental approach taken in this case study is not directly comparable to existing interactive approaches (cf. [21, 29, 41]).

## Background

This section introduces central process mining concepts used throughout this paper. First, we introduce event data in Sect. 3.1. Second, we introduce concurrency-aware process execution variants and related concepts in Sect. 3.2. Finally, we briefly introduce process models in Sect. 3.3.

### Event Data

Event data are the primary input for many process mining techniques as they describe the execution of processes. Table 1 shows an example of the event log subject to this case study. Each row represents an event. For example, the first event describes that the activity “General Physical Examination (GPE)” was executed on 15/05/2017 for the process execution with case ID 25480. Note that in this specific example, the case ID corresponds to the patient ID. Thus, each patient represents an individual process execution.

We refer to all events having an identical case ID as process execution or *trace*. For example, consider the events with case ID 47777. The first recorded event states that a general physical examination (GPE) was performed on the patient on 17/07/2017. Eight days later, on 25/07/2017, various examinations were performed: CT scan of the patient’s chest (CTC), Liver Biopsy (LiB), and various lab tests, i.e., Calcium (Cre), Glucose (Glu), Magnesium (Mag), and Creatinine (Cre).

The example trace above exhibits *partially ordered event data*, i.e., various events have identical timestamps within a trace. Note that most process mining techniques assume that events of a trace are totally ordered [13, 42], i.e., the events can be sequentially aligned according to their timestamps. However, partially ordered event data is a regular phenomenon in healthcare, as discussed in Sect. 1.^2^ Often, the timestamps of events are coarse; for example, only the day but not the exact time is recorded due to manual entry later, for instance, a nurse entering an X-ray execution at the end of a shift in an information system. Thus, we assume partially ordered event data in this case study. Thereupon, we propose in Sect. 4.2 a novel method, similar to [45], to integrate order relations derived from domain knowledge into partially ordered event data to improve the poor informative value of coarse timestamps.

### Trace Variants

Trace variants (hereinafter referred to simply as variants) are an essential concept in process mining. Variants group traces with identical order relationships among their activities. Therefore, variants are crucial in various process mining tasks that focus on control flow aspects of activities. In general, variants facilitate handling vast amounts of traces in event logs.

**Table 1:** Excerpt of the event log covering the treatment of lung cancer patients; each row represents a unique event

Case ID	Activity label	Activity category	Timestamp	. . .
25480	General Physical Examination (GPE)	–	15/05/2017	. . .
25480	Creatinine (Cre)	Examination	15/05/2017	. . .
25480	Calcium (Cal)	Examination	15/05/2017	. . .
25480	Glucose (Glu)	Examination	15/05/2017	. . .
25480	Magnesium (Mag)	Examination	15/05/2017	. . .
25480	Chest X-ray (ChX)	Examination	15/05/2017	. . .
25480	Spirometry (Spi)	Examination	25/05/2017	. . .
25480	General Physical Examination (GPE)	–	01/06/2017	. . .
47777	General Physical Examination (GPE)	–	17/07/2017	. . .
47777	CT Chest (CTC)	Examination	25/07/2017	. . .
47777	Calcium (Cal)	Examination	25/07/2017	. . .
47777	Glucose (Glu)	Examination	25/07/2017	. . .
47777	Magnesium (Mag)	Examination	25/07/2017	. . .
47777	Creatinine (Cre)	Examination	25/07/2017	. . .
47777	Liver Biopsy (LiB)	Examination	25/07/2017	. . .
47777	Electrocardiogram (Elc)	Examination	18/08/2017	. . .
47777	Spirometry (Spi)	Examination	18/08/2017	. . .
47777	Excision of lung and bronchus (ELB)	Surgery	01/09/2017	. . .
47777	Computer aided surgery (CAS)	Surgery	01/09/2017	. . .
47777	Other non-operative procedure (ONOP)	Treatment	10/09/2017	. . .
47777	Other non-operative procedure (ONOP)	Treatment	10/09/2017	. . .
47777	General Physical Examination (GPE)	–	15/09/2017	. . .
47777	Calcium (Cal)	Examination	15/09/2017	. . .
47777	Glucose (Glu)	Examination	15/09/2017	. . .
47777	Magnesium (Mag)	Examination	15/09/2017	. . .
47777	Creatinine (Cre)	Examination	15/09/2017	. . .
40036	General Physical Examination (GPE)	–	01/07/2017	. . .
40036	Calcium (Cal)	Examination	01/07/2017	. . .
. . .	. . .	. . .	. . .	. . .

**Fig. 2:**
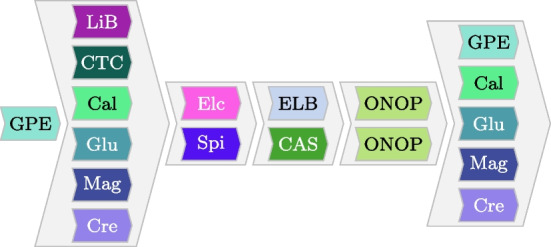
Concurrency-aware variant describing trace 47777 (cf. Table 1)

Since we focus in this use case study on partially ordered event data, we use *concurrency-aware variants*, which were recently introduced [46]. In the remainder of this paper, we refer to concurrency-aware variants as variants. Figure 2 illustrates a variant that describes the trace with case ID 47777.

In general, vertically aligned activities indicate that they were performed simultaneously or overlapped, while horizontally aligned activities represent consecutive execution. Thus, for the given example in Fig. 2, all activities aligned vertically are executed on the same day. Moreover, since days are the bottom granularity of the provided timestamps, i.e., there is no information about the exact time, all vertically aligned activities do not have an execution order; they are considered to be executed in parallel. Using different colors in the variant visualization facilitates distinguishing and identifying various activities.

First, the activity General Physical Examination (GPE) is executed. Next, the activities LiB, CTC, Cal, Glu, Mag, and Cre are executed in parallel since all have identical timestamps in trace 47777. After these six activities, Elc and Spi are executed in parallel, followed by ELB and CAS being executed in parallel and two parallel executions of activity ONOP. Eventually, the activities GPE, Cal, Glu, Mag, and Cre are executed in parallel.

In short, variants are essential and allow for clustering various traces with identical order relationships among their activities. Since partially ordered event data are a regular phenomenon in the healthcare domain, we consider concurrency-aware variants in this paper. For a detailed introduction into concurrency-aware variants and their computation, we refer to [46, 47].

### Process Models

Process models allow us to formalize processes. Various formalisms exist, for example, BPMN models [48], Petri nets/workflow nets [49, 50], and process trees [13]. Most process model formalisms focus on modeling the control flow of the involved activities.^3^ These models show optional activities, repetition of activities, decision points, and parallel execution branches in processes.

**Fig. 3:**
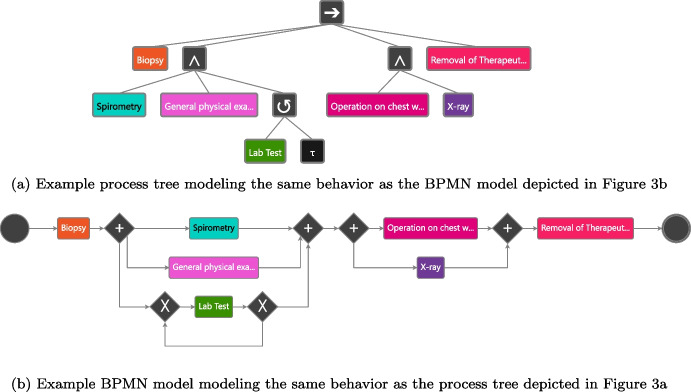
Example process model once specified as a process tree and once as a BPMN model

Figure 3a provides an example process tree that models a lung cancer treatment process. The root node represents a sequence operator ($\rightarrow$); thus, its subtrees are thus executed according to their order. Hence, activity Biopsy is executed first. Next, a subtree with a parallel operator ($\wedge$) is executed. Thus, all its subtrees/activities are executed parallel respectively in any order, i.e., Spirometry, General physical examination and Lab Test. Note that Lab Test is located under a loop operator ($\circlearrowleft$); thus, Lab test can be executed multiple times but at least once. Next, Operation on chest wall and X-ray are executed in parallel. Finally, the activity Removal of Therapeutic Appliances is executed. Figure 3b shows the described process as a BPMN model.

We refer to [13] for an extensive introduction to process trees and [48, 51] for an introduction to BPMN. We focus on process trees in this paper because the interactive/incremental process discovery techniques used, produce process trees. However, as exemplified in Fig. 3b, such process trees can also be easily transformed into other model formalisms, e.g., BPMN models or Petri nets. In conclusion, process models such as process trees allow us to model the control flow of the activities within a process and are an essential artifact in process mining that are used for many subsequent analysis tasks.

## Interactive Process Discovery Tool Cortado

This section presents the process mining software tool Cortado, which we use to analyze the event log that is the subject of this case study. Cortado is an open-source software [31], featuring domain-knowledge-utilizing process discovery approaches [24]. Further, it features event data processing and exploration techniques. Thus, Cortado combines all the required techniques to analyze event data interactively. In the remainder of this section, we briefly overview its functionalities in Sect. 4.1. Further, Sect. 4.2 introduces a novel function for manipulating variants based on domain knowledge that we developed explicitly in the context of this case study.

**Fig. 4:**
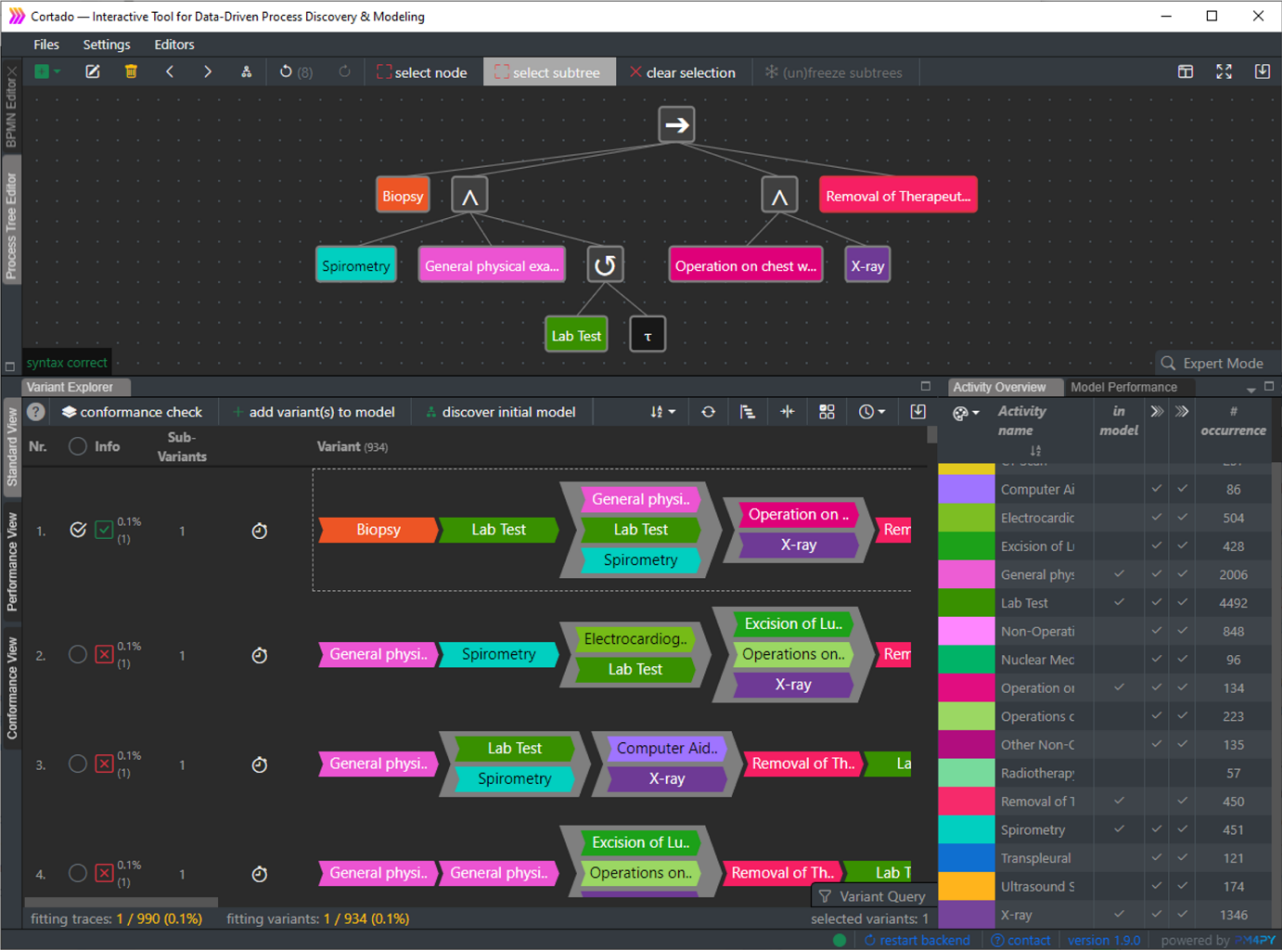
Screenshot of the open-source process mining tool Cortado, which is used for analyzing the event data subject to this case study

### Overview Existing Functionalities

Cortado features techniques covering the full interactive process discovery cycle as illustrated in Fig. 1. Subsequently, we provide a brief overview of existing functionality relevant to this case study. For a complete overview of Cortado’s functionality, we refer to [31].

Central to *event data exploration* are variants as introduced in Sect. 3.2 and exemplified in Fig. 2. Cortado visualizes all variants from a given event log in a variant explorer allowing manual exploration by users. Figure 4 shows a screenshot of Cortado. In the lower part of the screenshot, the variant explorer is visible. To handle vast amounts of variants, a query language [52] allows targeting variants to be found using specified activity patterns. In addition, Cortado features frequent pattern mining techniques for variants and variant clustering algorithms. Regarding *event data filtering*, Cortado offers various approaches that are combined with the techniques described above; for example, users can utilize the query language or frequent variant pattern mining to filter event data respectively variants.

From selected variants, users can discover an initial model. Cortado offers a process model editor supporting BPMN models and process trees, as shown in Fig. 4. Such an initial model can be incrementally extended by non-supported process behavior, i.e., variants the process model does not yet describe. To compare process models with the provided event data, Cortado offers *conformance checking* [53] functionality. For instance, variants can be filtered according to their conformance level with the current process model.

### Integrating Domain Knowledge into Variants

This section presents the new functionality *variant sequentialization* in Cortado that we implemented in the context of this case study. Recall the event log shown in Table 1. As discussed, we consider partially ordered event data, i.e., multiple events of a trace may have identical timestamps and are thus executed in parallel. Reconsider the variant shown in Fig. 2 that indicates various activities being executed concurrently. However, since the events’ timestamps have a coarse temporal granularity, i.e., the bottom granularity is days and no time is recorded, their temporal information is rather inaccurate.

The partially ordered event data indicates that various activities from a trace happen simultaneously (cf. Fig. 2), i.e., on the same day. However, certain activities that happen on the same day have some order relations if considering medical domain knowledge. For instance, the variant visualized in Fig. 2 indicates that activities Liver Biopsy (LiB) and CT Chest (CTC) are executed in parallel, i.e., in this specific case on the same day. Thus, when discovering a process model using this variant, the model would indicate that these activities happen in parallel respectively in any order. From a practical view, however, we know that the CT Chest (CTC) activity happens always before Liver Biopsy (LiB). Thus, we would like to integrate such domain knowledge into the variants.

**Fig. 5:**
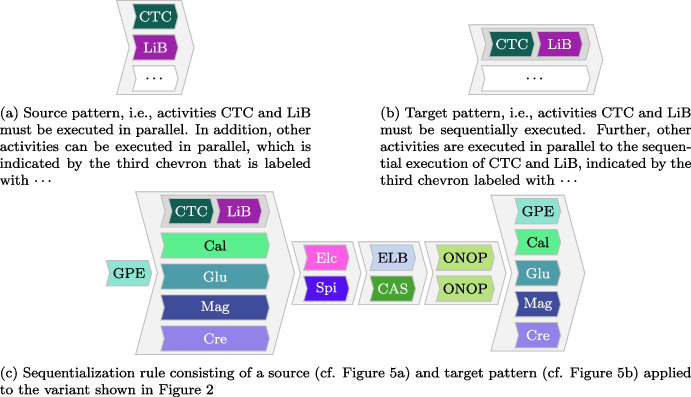
Example of the sequentialization feature allowing to sequentialize parallel execution of activities as indicated by variants based on domain knowledge, i.e., source and target patterns are defined by users

To this end, we implemented the *variant sequentializer*, allowing to integrate domain knowledge into variants. We allow to specify source and target patterns that specify fragments of variants. Figure 5a depicts an example source pattern for the given example. The exemplified source pattern matches any variant where activity CTC is in parallel to LiB; more activities can happen in parallel as indicated by the white-colored chevron labeled “$\cdots$.” The target pattern, depicted in Fig. 5b, specifies that activity CTC is executed before LiB; all other activities (i.e., white-colored chevron labeled “$\cdots$”) are executed in parallel to this sequential execution of CTC and LiB. Reconsider the variant depicted in Fig. 2. The source pattern depicted in Fig. 5a is present in this variant. Thus, when applying the sequentialization rule, consisting of the source (cf. Fig. 5a) and target pattern (cf. Fig. 5b), to the variant, we obtain the modified variant as visualized in Fig. 5c.

The described *variant sequentialization* functionality allows users to integrate domain knowledge into variants, which are central for incremental process discovery. This is particularly valuable as the integration of such domain knowledge allows for the establishment of ordering relationships prior to the inclusion of this process behavior in a model. Further, reasoning on individual variants (representing patients with identical treatments in this case study) is often easier with non-process-mining experts than fixing such order relations within a process model. Since process models typically describe a large number of variants, the cognitive effort required to change such order relationships in process models is significantly higher, because more variants have to be taken into account at the same time in order to avoid entering incorrect orders that may not be applicable in all variants. Another aspect that must be considered when using variant sequentialization is the conformity of a discovered process model with the original event log. Suppose a process model discovered from sequentialized variants is compared with the original event log. In that case, deviations between the model and the event data might be detected as the original event log includes traces containing sequentialized activities with the same timestamp.

## Research Design

This section provides a comprehensive overview of the study conducted within the healthcare domain. Section 5.1 outlines the overarching research goal and introduces the specific process under analysis, while Sect. 5.2 details the methodology employed in the study.

### Research Goal and Setting

The primary aim of this research was to evaluate the suitability and effectiveness of incremental process discovery techniques in analyzing complex healthcare processes characterized by a high level of knowledge intensity. Our goal was to leverage these innovative techniques to develop a robust and reliable normative process model tailored to the intricate nature of healthcare procedures. In pursuit of this objective, we aimed to integrate the expertise of healthcare professionals with detailed event data documenting patient treatments. By combining these two sources of information, our aim was to create a comprehensive process model that accurately reflects procedural insights while capturing the dynamic nature of patient care as evidenced by real-world treatment data.

The study was conducted within a medium-sized public hospital in Italy. The dataset used in the study encompassed detailed information on the primary treatments administered to hospitalized patients diagnosed with lung cancer over the course of a year. Lung cancer treatment is inherently complex and necessitates a multidisciplinary approach, involving collaboration among professionals with diverse specialties. The Italian Association of Medical Oncology guidelines [54] assist clinicians in treating such diseases. More in detail, each lung cancer patient undergoes a set of activities that include diagnostic activities (e.g., X-ray, CT scan, spirometry, bronchoscopy) to confirm the diagnosis and assess the extent of the disease, as well as surgery and follow-up activities. However, given the complexity of the disease and the variety of treatments, many special and ad hoc care pathways may exist.

The study was conducted in close collaboration with a multidisciplinary medical team comprising the head of the thoracic surgery department, an oncologist, and a doctor and nurse from the pneumology department, all of whom specialize in providing specialized care to patients with lung cancer. The head of thoracic surgery holds expertise in performing surgical interventions for lung cancer and conditions affecting the mediastinum, chest wall, and esophagus. The oncologist, on the other hand, specializes in diagnosing and treating various forms of cancer, playing a crucial role in the comprehensive care of patients. Additionally, the doctor and nurse in the pneumology department play an active role in delivering attentive care to individuals with respiratory diseases, ensuring holistic patient management.

### Research Methodology

The methodology applied for the study consists of two main phases: *event data extraction and initial preparation* and *interactive process discovery*, as shown in Fig. 1. In the following, we provide insights and details into these phases.

1. The first phase, *Event Data Extraction and Initial Preparation*, aimed to collect data from various hospital information systems and create an event log suitable for the analysis. This phase involved merging datasets, removing redundant or inconsistent information, and producing a single, coherent dataset that can be readily interpreted during the process discovery stage. For this phase, established techniques and approaches  [15, 42, 55, 56].
2. The second phase, *Interactive Process Discovery*, played a pivotal role in process analysis. Utilizing Cortado  [31] and its functionalities, this phase facilitated interactive exploration of variants, incremental process discovery, and analysis. The integration of domain knowledge from the medical team alongside event data was essential for ensuring the credibility of the findings. This phase, executed cyclically as needed, comprised three sub-phases (cf. Fig. 1).
  1. The *Event Data Processing and Exploration* phase aimed to explore, organize, sort, and select variants for the subsequent incremental process discovery phase—as described before, variants are central to incremental process discovery. Cortado’s capabilities like variant visualizations (cf. Sect. 3.2) and variant querying [52] supported the research team in this phase, along with variant filtering and editing based on domain knowledge. For example, consider the variant sequentialization feature introduced in Sect. 4.2.
  2. The *Incremental Process Discovery* phase aimed to discover the model of the patient treatment process gradually, integrating selected variants into the model using corresponding algorithms  [31]. During this phase, the research team guided the algorithm, for example, by freezing certain parts of the process model  [57].^4^ Further, the research team manually edited parts of the process model based on the domain knowledge from the medical team.
  3. In the *Evaluation and Analysis* phase, the research team assessed the incrementally extended process model from the previous phase. Conformance checking techniques  [53] were employed to evaluate how well the model aligned with provided and modified event data and domain knowledge, identifying potential gaps or inconsistencies that required further iterations of the interactive process discovery phase. This iterative process ensured continuous refinement and improvement of the process model. At the end, the model underwent further validation with the medical team.

Throughout the study, the medical team played an integral role. Particularly, we organized two meetings: one prior to commencing the Event Data Extraction and Initial Preparation phase, and another following the Evaluation and Analysis phase. The pre-study meeting aimed to contextualize the research by obtaining pertinent information about the process, the organization, and clinical guidelines. Conversely, the follow-up meeting sought feedback on the innovative interactive process discovery approach enabled by Cortado and its resulting output. Additionally, during the follow-up session, we prompted the team to qualitatively compare Cortado’s output with that of Inductive Miner (IM)  [58], a widely used automated technique, especially in healthcare  [1]. Both meetings, each lasting 2 h, were conducted online via video conference, involving two researchers and the medical team. One researcher directed the discussion with general questions, while the other moderated. Transcriptions of the discussions were collected and analyzed alongside hospital procedures and clinical guidelines documents to ensure triangulation of information. In addition, during the Interactive Process Discovery phase, the medical team provided ongoing support and feedback.

This study uses a different approach to process mining compared to traditional sequential methods like PM^2^ [55] and the healthcare-specific method presented in [15]. Rather than following a strictly sequential process of data preparation, process discovery, and evaluation, our approach utilizes a cyclic procedure that allows for revisiting and re-executing previous phases or sub-phases as needed. Refer to Fig. 1 for a visual representation.

## Implementation and Results

In this section, we outline the actions we took during each stage of the methodology for the lung cancer study. Section 6.1 covers the outcomes of the initial phase, where we extracted and prepared event data. Meanwhile, Sect. 6.2 explains the subsequent interactive process discovery phase approach and performed actions.

**Fig. 6:**
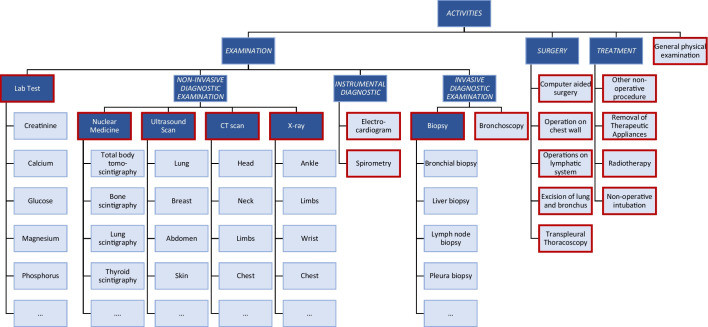
Overview of the activity hierarchy from the event log analyzed in this case study. Activities highlighted in light blue are from the extracted event log, while activities highlighted in dark blue represent abstracted activities, i.e., activities below dark blue activities are a specialization. Activities outlined in red have been identified as the correct abstraction level for the analysis objectives and will be considered in further analysis

### Event Data Extraction and Initial Preparation

We gathered event data from two hospital information systems: the electronic medical record (EMR) and the radiology information system (RIS). The EMR is a comprehensive repository that stores information about all inpatient episodes that take place within the hospital. It provides a detailed account of the patient’s medical history, diagnoses, treatments, and other relevant data. Similarly, the RIS is a dedicated platform for managing and organizing information related to radiology activities performed within the hospital. It includes details about imaging orders, results, reports, and scheduling. These systems store valuable information, which we combined into a single event log.

The initial event log consists of 998 cases corresponding to 998 different patients, 45 different activities, and more than 40,000 events. Note that the timestamp was recorded at the date level for each event; no time information is available, as exemplified in Table 1.

We initially performed a preliminary cleaning to resolve obvious data quality problems, including the following actions.

- *Outliers and incomplete cases removal:* We removed eight cases with incorrect activity time records and missing relevant attribute values.
- *Event abstraction:* The event log contains various activities categorized into different levels of detail. An overview of the activity hierarchy is shown in Fig. 6. To achieve our analysis goals, we relabel low-level activities, i.e., we generalize activities. For instance, we relabeled lab test-related activities, such as glucose, potassium, and creatinine, into one macro-activity named “Lab Test.” Similarly, we combined radiological activities, like CT abdomen, CT brain, wrist X-ray, and knee X-ray, into representative activities named “X-ray” and “CT scan.” The described approach reduced the number of activity labels from 45 to 19, as shown in Fig. 6, i.e., the activities outlined in red were selected as the optimal level of abstraction.
- *Elimination of lesser activities:* We removed less significant activities not directly related to treating lung cancer disease, for instance, eye lens operation, bone excision, and ovariectomy. These activities are due to the onset of comorbidity in the patient, i.e., another diagnostically definable syndrome that occurs in addition to an underlying disease. Therefore, for our analysis, we decided to abstract them.

The refined event log consists of more than 14,000 events, 990 patient cases, and 19 types of activities. The remaining activities are outlined in red in Fig. 6. As expected, the process heterogeneity is very high with 934 different variants out of 990 cases that makes process modeling and analysis extremely complex as almost each patient treatment is unique regarding the ordering of performed activities.

### Interactive Process Discovery

In this section, we explain the actions performed during three main sub-phases of the *Interactive Process Discovery* phase (cf. Fig. 1) and report the corresponding results.

#### Event Data Processing and Exploration

By using the query language [52] implemented in Cortado, we initially explored in detail the numerous variants in the event log to investigate their main characteristics and remove any outliers that could not be detected in the previous step, i.e., the *Event Data Extraction and Initial Preparation* phase. It should be noted that in this stage, outliers are not anomalies within the process or infrequent behaviors; rather, they manifest as truncated or incomplete traces resulting from errors in data entry or extraction. This allowed us to partially reduce the complexity of the event data and improve the quality of the resulting process model.

Specifically, we investigated the frequency and the number of activities of which each variant is composed. For example, we noted that the most frequent variants (with an occurrence of 11 and 9 cases, respectively) consist of only one activity, sometimes repeated multiple times. In addition, 0.4% of variants consist of a maximum of three activities, while 0.2% of variants comprise up to six activities of only two types. As confirmed by the medical team, these are problematic variants that do not reflect the actual execution of the process but result from poor data quality. Indeed, it is implausible for a patient to be hospitalized for only one or two examinations. In general, hospitalization involves a series of tests, treatments, visits, and sometimes surgery, while single examinations are performed in an outpatient setting. These outliers may be due to registration errors or non-registration by hospital staff. Therefore, we decided to remove such variants.

**Fig. 7:**
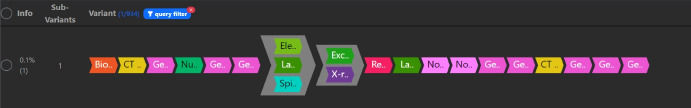
Screenshot of Cortado showing a variant that indicates an inconsistent ordering of activities, i.e., “Biopsy” (i.e., the first activity of the visualized variant) is executed before “CT scan,” i.e., yellow highlighted activities


Furthermore, we identified and removed incorrect variants, i.e., those with a sequence of activities that did not comply with the clinical guidelines and physicians’ expertise and could lead to inappropriate behaviors. For example, the medical staff suggests that at the beginning of the lung cancer treatment (diagnostic phase), invasive diagnostic procedures (for example, bronchoscopy or biopsy) must be executed after non-invasive diagnostic exams (for instance, X-ray and CT scan) to confirm the diagnosis and evaluate the extent of the disease. However, the event log contained variants in which this relationship is not respected. Figure 7 shows an example of the variants mentioned above, where a biopsy was performed before a non-invasive diagnostic procedure, i.e., a CT scan. The corresponding query that we use to identify and eliminate such inaccurate variants is shown in Listing 1. These incorrect variants may be due to registration errors or delayed registration that introduce noise in the dataset. As the presence of these behaviors may be undesirable as they may affect the reliability of the results and the subsequent process analysis, we decided to remove such variants.

Following this exploration and filtering phase, we removed 4% of the variants.

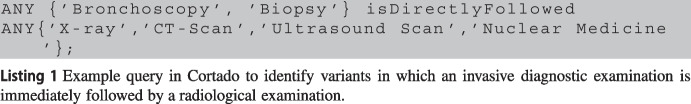

**Fig. 8:**
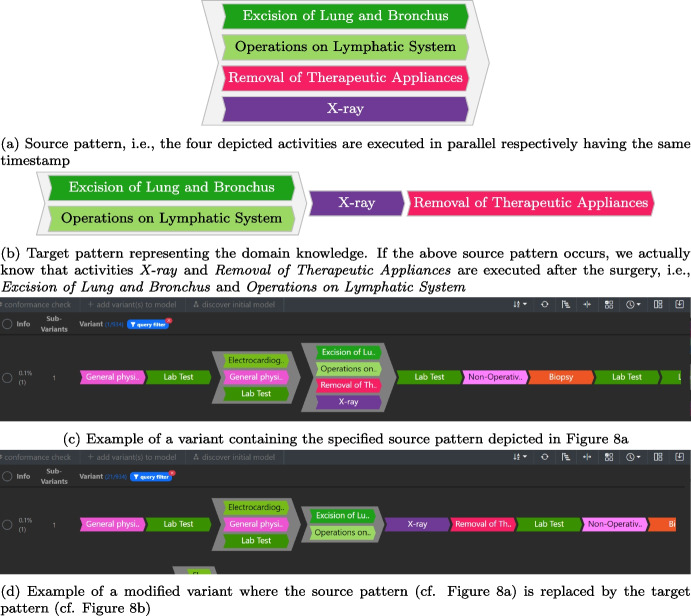
Example of an actual source target pattern pair for sequentializing variants and its application to a variant from the analyzed event log

Subsequently, by using the *variant sequentialization* function (cf. Sect. 4.2), we integrated domain knowledge into imprecise variants that are those variants consisting of several activities that occur in parallel but should follow a specific execution order, according to the guidelines and understanding of the medical staff. For example, in the surgery phase of lung cancer treatment, the patient generally undergoes the following activities: instrumental examinations to assess their operability, surgery, an X-ray, and, if present, removing the therapeutic device. These activities should occur sequentially; however, even if they are performed on the same day, we only observe the identical timestamps in the event log due to the coarse bottom granularity of days. For instance, consider the variant depicted in Fig. 8c as an example. The variant indicates that removing the therapy device could be performed before its installation during the surgery, as both activities are modeled in parallel. Thus, using this variant for process discovery results in an imprecise process model allowing for wrong behavior. By exploiting the variant sequentialization functionality in Cortado (cf. Sect. 4.2) and the knowledge of the medical staff, we manipulate these variants. Figure 8a depicts the described source pattern, while Fig. 8b depicts the corresponding target pattern. When applying this sequentialization rule to the variant shown in Fig. 8c, we obtain the sequentialized variant depicted in Fig. 8d. We implemented approximately 20 sequentialization rules, although we do not detail each one here.

#### Incremental Process Discovery

In this phase, after eliminating or refining variants plagued by data incompleteness and noise stemming from poor quality, we incrementally shaped the process to establish a benchmark regulatory model for lung cancer patient treatment. The incremental discovery process was conducted by using Cortado, wherein additional variants were gradually incorporated into the initial model. Any inaccuracies were resolved manually during each iteration by editing the model directly. When necessary, we re-performed the *Event Data Processing and Exploration* phase to eliminate inconsistencies further.

**Fig. 9:**
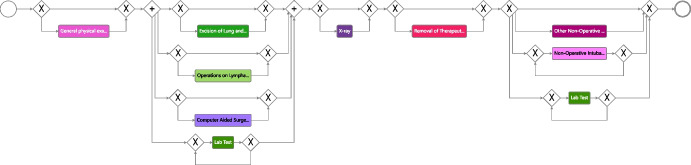
BPMN model describing the 15 most frequent variants

We started with an initial model that comprised only the variants devoid of diagnostic activities in the initial phase, accounting for about 0.15% of the variants, as shown in Fig. 9. It is worth noting that these particular variants represent the most common occurrences. This strategic approach was adopted to map the journey of hospitalized patients undergoing diagnostic procedures outside the hospital, either in outpatient settings or within private healthcare facilities. The initial process model contains three stages that make up the process.

1. The diagnosis stage comprises just the activity *general physical examination* describing the first visit with the physician.
2. The *surgery* stage contains the activities *Excision of Lung and Bronchus*, *Operations on Lymphatic System*, *Computer Aided Surgery*, *Lab Test*, *X-ray*, and *Removal of Therapeutic Appliances*.
3. The last stage denoted *follow-up* comprises the activities *Other Non-Operative Procedures*, *Non-Operative Intubation*, or further *Lab Tests*.

Building upon the initial model, we decided to incorporate shorter-length variants that encompassed diagnostic activities and, involved one surgical procedure per case into the model, constituting 20% of the overall variants. This helped us understand how the process changes once diagnostic tests are introduced and in which phase of the process these changes occur.

**Fig. 10:**
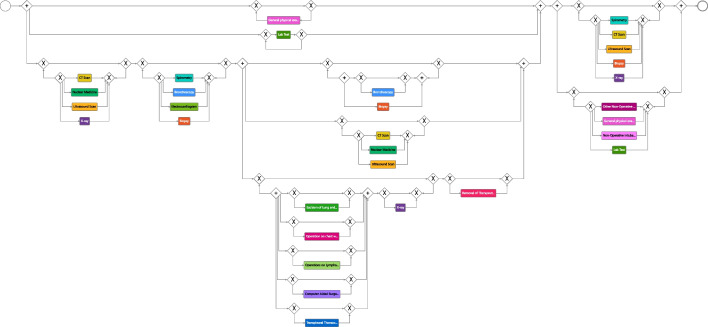
Intermediate BPMN model depicting treatment options for lung cancer patients undergoing surgical treatment. The model illustrates the sequential relationship between diagnostic and surgical phases, while highlighting the diverse activities involved in the follow-up phase, which may be repeated based on individual patient reactions and health conditions

At this point, we implemented two main manual adjustments to enhance the model’s clarity and reliability. First, we introduced a loop structure to handle diagnostic and follow-up activities as the number and type of examinations vary from patient to patient. Then, we restructured the model to include the *general physical examination* and *lab test* activities in parallel with the diagnostic and surgical activities because they are key activities that may be repeated frequently during the treatment process. Next, we added the remaining intricate variants (about 40% of the variants), encompassing diagnostic activities as well as various types of surgical procedures, as shown in Fig. 10. The model shows a sequentiality between the activities of the diagnostic phase and those of the surgical phase. In contrast, the follow-up phase involves a wide variety of activities that can be repeated multiple times based on the health condition and each patient’s reaction to the surgery. Furthermore, it can be noted that for more complex cases, additional radiological examinations and interventions were also required during the surgical phase.

**Fig. 11:**
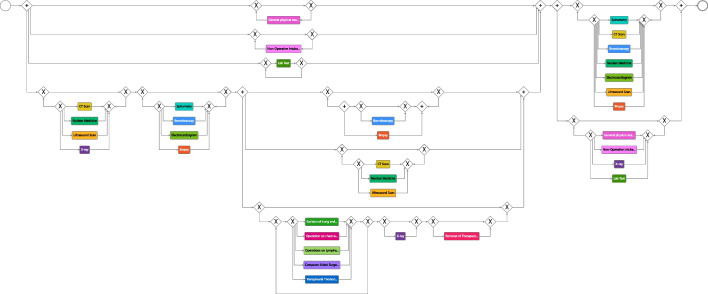
Final process model that describes the various options for lung cancer patient treatments. Notably, the diagnostic and surgical phases follow a well-defined sequence. In cases of complexity, the surgical phase may require supplementary radiological examinations and interventions. Conversely, the follow-up phase is dependent on the physician’s expertise and the individual patient’s condition, offering flexibility for the repetition of various treatment types across different patients or for a single patient

As a final step, before adding the remaining variants related to non-surgical hospitalized patients (about 40% of the overall variants), we froze all activities concerning the surgical phase in the model (roughly a third of the activities), so that there were no changes in this part of the process when continuing with incremental process discovery. To do so, we made use of the freezing functionality [57] implemented in Cortado. Figure 11 depicts the final discovered process model based on the event data and the provided domain knowledge. The resulting model describes all suitable variants of the event log and shows the following:

- The diagnostic and surgical phases have a more structured and defined sequence, as suggested by the guidelines.
- The follow-up phase is dependent on the physician’s experience and the patient’s condition.
- There are different types of treatments that can be performed multiple times for a single patient or for different patients.

#### Evaluation and Analysis

During the previous phase, it was possible to track in Cortado the accuracy of the discovered model with respect to the event log. The fitness value, which started at 30% during the initial iterations, gradually increased as more variants were included in the model. With modifications to the model, the indicator was raised to 70% and eventually reached 100% by the end of the phase, indicating a high level of conformance w.r.t. to the event log that was incrementally processed.

**Fig. 12:**
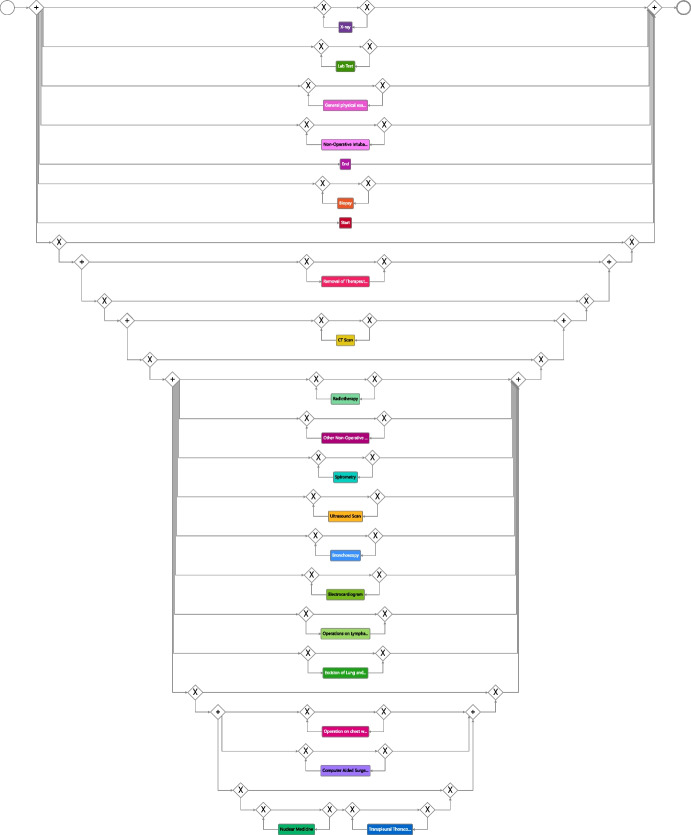
Process model discovered by the conventional process discovery algorithm Inductive Miner [58], with default parameters. The model places the main activities in parallel, which results in a loss of sequentiality between the macro phases of diagnostic, surgery, and follow-up. This lack of proper sequencing leads to improper behaviors, including the incorrect positioning of non-invasive and invasive diagnostic examinations

After completing multiple iterations of the interactive process discovery phase (cf. Fig. 1), we engaged in discussions with the medical team regarding the functionalities of Cortado. Furthermore, we requested all team members to provide qualitative evaluations (high, medium, low) for the models generated by Cortado (cf. Fig. 11) and IM (cf. Fig. 12), as outlined in Sect. 5.2. The evaluation focused on three aspects: model comprehensibility, adherence to guidelines, and simplicity in terms of represented elements. In employing IM, we used as input the event log obtained downstream of the *Event Data Processing and Exploration* phase, to make the qualitative/manual comparison of models fairer.

The medical team appreciated Cortado’s functionality related to interactive data exploration/manipulation. Specifically, two members highlighted the challenge of working with large amounts of patient data that are often dirty and unreliable, making it difficult to extract useful insights without dedicated tools. Therefore, they found it valuable to explore data and decide what to manipulate or filter out as deemed incorrect to obtain reliable and practical information. In addition, the interactivity of process discovery and the incremental approach was also seen as positive aspects. Indeed, a team member said that “[...] what is even more value-added is to be able to incrementally model the process and decide what to include, freeze or modify in the map [...], exploiting our knowledge.” To summarize, the active participation of the expert was reported by all team members as essential, as it helped to fill in the data gaps and provided valuable insights to enhance the quality of the model. However, during the follow-up meeting, a critical point was raised regarding using interactive data exploration and incremental process modeling features. It was highlighted that prior knowledge of fundamental process mining concepts and modeling languages is necessary to leverage these features entirely. Alternatively, the support of an experienced analyst would be beneficial in ensuring that these capabilities are utilized to their full potential. For example, a team member pointed out that “[...] I know neither petri nets nor decision trees, and I would not be able to conduct the analysis alone [...].”

Regarding the process models, the medical team found the process model created interactively with Cortado to be more comprehensible and consistent with the guidelines, although quite complex in terms of the number of elements. The process structure is clearly visible, and any non-compliances, due to low data quality, were removed during the modeling phase. On the other hand, the model produced by IM was considered confusing (e.g., one team member even expressed uncertainty regarding the interpretation of the IM model, saying “Is this the process for lung cancer patients?”) and presented improper behaviors that would require manual modifications, such as the position of non-invasive diagnostic examinations and invasive diagnostic examinations. Overall, the follow-up meeting provided valuable insights into the medical team’s perspectives on the new interactive approach enabled by Cortado and the process model produced.

## Discussion

This section discusses the presented case study. We will highlight the lessons learned, practical implications, and limitations and outline potential future work. In short, our findings indicate that interactive process discovery methods can effectively tackle the common challenges faced in the healthcare domain, such as complex and variable processes and low-quality data.

### Lessons Learned

Contained within this section, we have identified three crucial lessons gleaned from the presented case study.

#### Domain Experts

One lesson learned is that domain experts’ involvement is crucial for the success of any data-driven process modeling project. For example, domain experts improve the process model’s accuracy by adding tacit details not present in event logs. Furthermore, the involvement of domain experts plays a pivotal role in rectifying significant shortcomings or errors within a dataset. Take, for instance, a scenario highlighted in our case study where clinical guidelines prescribe a specific sequence for examinations. If this chronological order is not readily discernible within the dataset due to the absence of temporal information, domain experts can step in to address this issue. It’s important to note that this is not a process anomaly, but rather underscores a challenge related to data quality. In such situations, domain expertise becomes indispensable, seamlessly integrating itself into the process by identifying errors stemming from data quality issues that go beyond mere process anomalies or occasional irregularities.

The importance of domain experts was evident in both the *Event Data Processing and Exploration* phase, where experts provided input on what to filter out and manipulate, and in the *Incremental Process Discovery* phase, where they suggested which variants to add to the model and which changes to make to ensure compliance with guidelines. Establishing a guidelines-conformance model is indispensable for guaranteeing that physicians’ care practices are firmly grounded in scientific evidence. In summary, this case study contributes to the open problem of utilizing domain knowledge [30] in process mining by showcasing a concrete approach using the tool Cortado, allowing the utilization of domain expertise.

#### Blending of Event Data Processing and Process Discovery

Another lesson learned is that data exploration and especially manipulation must be integral to the process discovery phase and cannot be entirely automated. In our case study, many issues in the event log emerged during the *Event Data Processing and Exploration* phase, despite a preliminary data preparation. Some of these issues were easily recognizable (e.g., partial traces of a single activity), while others required domain knowledge (e.g., traces with activity sequences that did not comply with guidelines). In this context, the strong focus on variants in Cortado is also beneficial to help domain experts better understand the behavior in the event data. Also in [40], the authors report that “domain experts typically think of processes on a case level.” Therefore, the use of variant visualizations that summarize similar process executions is of great benefit in communicating with domain experts and fosters them to apply their domain knowledge, for instance, by the variant sequentialization functionality introduced in Sect. 4.2.

Further, we learned that data exploration, process discovery, and analysis could not be performed sequentially, as often happens in traditional process mining methodologies [15, 55]. Sometimes, it may be necessary to go back and repeat intermediate steps to improve/correct any inconsistencies. In our case study, we encountered additional data issues during the incremental discovery of the process model, which necessitated revisiting the previous phase to carry out the *variant sequentialization* task.

#### Incremental Process Discovery

Incremental process discovery has shown to be more effective in producing accurate models that comply with guidelines, particularly when faced with data quality issues compared to automated process discovery. This incremental approach allows the analysis and selection of suitable variants to include in the final model, identifies and resolves any inconsistencies, and builds confidence in the model. Conversely, wholly automated discovery techniques often produce confusing and unreliable process models, as revealed in the follow-up meeting with the medical team. In addition, these models require further manual modifications, making the task laborious.

A challenge encountered during the case study was that using interactive and incremental discovery techniques requires knowledge of process mining and modeling languages. This challenge confirms the findings by [59], where the authors identify poor analytical skills from people as a critical challenge in applying process mining in organizations. Further, also in [40], the authors identify the lack of process model formalism skills of domain experts as a challenge. The team involved in the study, and more generally, medical teams, do not have this knowledge. Therefore, the support of an experienced process mining analyst was essential, along with brief training. However, the incremental approach adopted in this case study partially addresses this issue as process models evolve when more variants are added. Therefore, the incremental changes are more accessible for domain experts in the process model and provide a basis for discussion, as often only a few parts of a process model change per iteration.

### Practical Implications

From a practical point of view, our study provides evidence of how an incremental and interactive approach can help managers model healthcare processes while effectively addressing the challenges posed by high process variability and poor data quality. By utilizing their knowledge and the capabilities of Cortado, healthcare organizations can gain valuable insights into their business processes, even when faced with poor data quality. Specifically, they can quickly identify the correct process variants, eliminate wrong sequences, and obtain comprehensible and compliant process models, to make informed decisions and optimize process performance. In particular, easy-to-understand visualizations such as the variant visualization [46] implemented in Cortado make a significant contribution to this. These findings are of significant interest to healthcare managers and practitioners seeking to leverage data-driven approaches for process improvement in their organizations.

Establishing an accurate and guidelines-conformance model is indispensable for ensuring a standardized approach to patient care, firmly rooted in the most current evidence available at the time of its development. However, it is paramount to underscore the need for ongoing updates to the model, in light of evolving clinical research and trials, patient-centered research, and emerging technologies. Particularly, a model that adheres to guidelines may be considered a reasonable starting point for decision-making and care delivery. Clinical guidelines are meticulously crafted from the most robust and up-to-date research findings, guaranteeing that physicians provide care rooted in scientific evidence. This foundation significantly enhances diagnostic precision, treatment efficacy, and patient outcomes. Standardized models also play a pivotal role in mitigating the risk of medical errors. Adhering to well-defined guidelines helps avert errors like medication mishaps, inaccurate diagnoses, and unnecessary procedures, thus fostering a safer environment for patients. Furthermore, it furnishes a structured pathway that facilitates a systematic and informed approach to service (re-)design. Embracing guidelines compliance streamlines the resource allocation process during the planning phase, offering a clear roadmap for identifying and assigning the necessary resources—be it personnel and equipment. These allocations are based on the predetermined requirements outlined in the guidelines. This proactive approach minimizes resource wastage and optimizes efficiency, a crucial aspect of service planning that substantially contributes to cost-effectiveness and operational excellence.

### Limitations and Future Work

Despite the promising results of this study, some limitations should be considered. Firstly, the findings are context-specific and may not be easily generalizable to other healthcare settings or business contexts. Moreover, the validation of the results was limited to a single follow-up meeting with a small group of practitioners. Although this meeting provided valuable feedback on the developed model, a more comprehensive validation process would be necessary to ensure the robustness and reliability of the approach. To address these limitations and further develop the approach, we plan to conduct a structured user study involving a broader, more diverse group of healthcare professionals. This approach will enable us to evaluate the interactive approach quantitatively, identify additional insights, and explore potential challenges.

## Conclusion

This study demonstrated the effectiveness of interactive and incremental process mining techniques, combined with domain knowledge, in modeling and discovering knowledge-intensive processes in the healthcare sector. Our case study, which utilized a real dataset from an Italian hospital documenting the treatment of lung cancer patients, demonstrated the benefits of using Cortado in process discovery. Unlike other interactive and traditional process discovery techniques [13, 21], Cortado combines data exploration and processing with the modeling phase, leading to a more integrated and efficient approach. This approach helped experts and analysts identify frequent patterns and incorrect variants, reduce variability, and remove outliers, resulting in a clinically guideline-compliant and accurate process model.

From a scientific perspective, this case study contributes to the field of process mining and business process management by addressing two state-of-the-art challenges, i.e., determining a suitable level of details for process activities and augmenting process mining with common sense and expertise  [30]. Further, we developed a novel technique for integrating domain knowledge into variants by manipulating variants, i.e., sequentializing parallel variant patterns. This feature is especially valuable when facing high process variability and data quality problems, where variants are often incomplete or incorrect, negatively impacting results. Furthermore, the study answers the need for more effective approaches to discovering/modeling healthcare processes  [14] by providing empirical evidence on the effectiveness of the interactive process discovery approach in healthcare. Indeed, this study represents one of the first attempts to apply an innovative interactive method in a complex context like healthcare, highlighting the importance of such an approach in improving the efficiency and effectiveness of the process discovery process. Finally, the cyclical methodology proposed to conduct the case study enables analysts and experts to re-execute previous steps as needed, allowing for continuous improvement and refinement of the process model. This approach stands in contrast to traditional linear approaches to process mining, for example,  [55], which can be limiting in complex and dynamic settings like healthcare.

## Data Availability

The open-source software tool Cortado can be accessed online at https://github.com/cortado-tool/cortado. The event log used is not publicly accessible for data protection reasons.

## Footnotes

1 Also, outside of the healthcare domain, little evidence exists.

2 Other publicly available event logs outside the healthcare domain that capture actual processes likewise comprise partially ordered event data, for instance, cf. [43, 44]

3 For example, BPMN contains graphical elements to model data flows and organizational aspects such as resources [51].

4 For a detailed and technical introduction into this feature, we refer to [57]

## References

[1] Mans RS, van der Aalst WMP, Vanwersch RJB (2015) Process mining in healthcare. Springer, Cham. 10.1007/978-3-319-16071-9 10.1007/978-3-319-16071-9

[2] Adeyemi S, Demir E, Chaussalet T (2013) Towards an evidence-based decision making healthcare system management: modelling patient pathways to improve clinical outcomes. Decis Support Syst 55(1):117–125. 10.1016/j.dss.2012.12.039 10.1016/j.dss.2012.12.039

[3] Duma D, Aringhieri R (2017) Mining the patient flow through an emergency department to deal with overcrowding. In: Cappanera P, Li J, Matta A, Sahin E, Vandaele NJ, Visintin F (eds) Health care systems engineering, vol 210 of Springer proceedings in mathematics & statistics, Springer International Publishing, Cham, pp 49–59. 10.1007/978-3-319-66146-9_5

[4] Stefanini A, Aloini D, Benevento E, Dulmin R, Mininno V (2018) Performance analysis in emergency departments: a data-driven approach. Measur Bus Excell 22(2):130–145. 10.1108/MBE-07-2017-0040 10.1108/MBE-07-2017-0040

[5] Gjellebæk C, Svensson A, Bjørkquist C, Fladeby N, Grundén K (2020) Management challenges for future digitalization of healthcare services. Futures 124:102636. 10.1016/j.futures.2020.102636 10.1016/j.futures.2020.102636

[6] Kraus S, Schiavone F, Pluzhnikova A, Invernizzi AC (2021) Digital transformation in healthcare: analyzing the current state-of-research. J Bus Res 123:557–567. 10.1016/j.jbusres.2020.10.030 10.1016/j.jbusres.2020.10.030

[7] Häyrinen K, Saranto K, Nykänen P (2008) Definition, structure, content, use and impacts of electronic health records: a review of the research literature. Int J Med Informat 77(5):291–304. 10.1016/j.ijmedinf.2007.09.001 10.1016/j.ijmedinf.2007.09.001 17951106

[8] Tandon A, Dhir A, Islam AN, Mäntymäki M (2020) Blockchain in healthcare: a systematic literature review, synthesizing framework and future research agenda. Comput Ind 122. 10.1016/j.compind.2020.103290

[9] Bhatia M, Sood SK (2017) A comprehensive health assessment framework to facilitate IoT-assisted smart workouts: a predictive healthcare perspective. Comput Ind 92–93:50–66. 10.1016/j.compind.2017.06.009 10.1016/j.compind.2017.06.009

[10] Oh H, Rizo C, Enkin M, Jadad A (2005) What is eHealth (3): a systematic review of published definitions. J Med Int Res 7(1). 10.2196/jmir.7.1.e1 10.2196/jmir.7.1.e1 PMC1550636 15829471

[11] Craig J, Patterson V (2005) Introduction to the practice of telemedicine. J Telemed Telecare 11(1):3–9. 10.1177/1357633X0501100102 10.1177/1357633X0501100102 15829036

[12] Basile LJ, Carbonara N, Pellegrino R, Panniello U (2023) Business intelligence in the healthcare industry: the utilization of a data-driven approach to support clinical decision making. Technovation 120. 10.1016/j.technovation.2022.102482

[13] van der Aalst WMP (2016) Process mining: data science in action. Springer. 10.1007/978-3-662-49851-4 10.1007/978-3-662-49851-4

[14] Munoz-Gama J, Martin N, Fernandez-Llatas C, Johnson OA, Sepúlveda M, Helm E, Galvez-Yanjari V, Rojas E, Martinez-Millana A, Aloini D, Amantea IA, Andrews R, Arias M, Beerepoot I, Benevento E, Burattin A, Capurro D, Carmona J, Comuzzi M, Dalmas B, de La Fuente R, Di Francescomarino C, Di Ciccio C, Gatta R, Ghidini C, Gonzalez-Lopez F, Ibanez-Sanchez G, Klasky HB, Prima Kurniati A, Lu X, Mannhardt F, Mans R, Marcos M, Medeiros de Carvalho R, Pegoraro M, Poon SK, Pufahl L, Reijers HA, Remy S, Rinderle-Ma S, Sacchi L, Seoane F, Song M, Stefanini A, Sulis E, ter Hofstede AHM, Toussaint PJ, Traver V, Valero-Ramon Z, de van Weerd I, van der Aalst WMP, Vanwersch R, Weske M, Wynn MT, Zerbato F, (2022) Process mining for healthcare: characteristics and challenges. J Biomed Informat 127. 10.1016/j.jbi.2022.103994 10.1016/j.jbi.2022.103994 35104641

[15] Rebuge Á, Ferreira DR (2012) Business process analysis in healthcare environments: a methodology based on process mining. Inf Syst 37(2):99–116. 10.1016/j.is.2011.01.003 10.1016/j.is.2011.01.003

[16] Stefanini A, Aloini D, Benevento E, Dulmin R, Mininno V (2020) A process mining methodology for modeling unstructured processes. Knowl Process Manage 27(4):294–310. 10.1002/kpm.1649 10.1002/kpm.1649

[17] Di Ciccio C, Marrella A, Russo A (2015) Knowledge-intensive processes: characteristics, requirements and analysis of contemporary approaches. J Data Semantics 4(1):29–57. 10.1007/s13740-014-0038-4 10.1007/s13740-014-0038-4

[18] Martin N (2019) Using indoor location system data to enhance the quality of healthcare event logs: opportunities and challenges. In: Daniel F, Sheng QZ, Motahari H (eds) Business process management workshops, vol 342 of lecture notes in business information processing, Springer International Publishing, Cham, pp 226–238. 10.1007/978-3-030-11641-5_18

[19] Fernandez-Llatas C, Bayo JL, Martinez-Romero A, Benedi JM, Traver V (2016) Interactive pattern recognition in cardiovascular disease management. A process mining approach. In: 2016 IEEE-EMBS International conference on Biomedical and Health Informatics (BHI), IEEE, pp 348–351. 10.1109/BHI.2016.7455906

[20] Vanbrabant L, Martin N, Ramaekers K, Braekers K (2019) Quality of input data in emergency department simulations: framework and assessment techniques. Simul Model Pract Theory 91:83–101. 10.1016/j.simpat.2018.12.002 10.1016/j.simpat.2018.12.002

[21] Benevento E, Aloini D, van der Aalst WMP (2022) How can interactive process discovery address data quality issues in real business settings? Evidence from a case study in healthcare. J Biomed Informat 130. 10.1016/j.jbi.2022.104083 10.1016/j.jbi.2022.104083 35504544

[22] Fernandez-Llatas C (2021) (ed) Interactive process mining in healthcare, Health Informatics, Springer. 10.1007/978-3-030-53993-1

[23] Andrews R, van Dun C, Wynn MT, Kratsch W, Röglinger M, ter Hofstede A (2020) Quality-informed semi-automated event log generation for process mining. Decis Support Syst 132:113265. 10.1016/j.dss.2020.113265

[24] Schuster D, van Zelst SJ, van der Aalst WMP (2022) Utilizing domain knowledge in data-driven process discovery: a literature review. Comput Ind 137. 10.1016/j.compind.2022.103612

[25] Augusto A, Conforti R, Dumas M, La Rosa M, Maggi FM, Marrella A, Mecella M, Soo A (2019) Automated discovery of process models from event logs: review and benchmark. IEEE Trans Knowl Data Eng 31(4):686–705. 10.1109/TKDE.2018.2841877 10.1109/TKDE.2018.2841877

[26] de Weerdt J, de Backer M, Vanthienen J, Baesens B (2012) A multi-dimensional quality assessment of state-of-the-art process discovery algorithms using real-life event logs. Inf Syst 37(7):654–676. 10.1016/j.is.2012.02.004 10.1016/j.is.2012.02.004

[27] van Dongen BF, Alves de Medeiros AK, Wen L (2009) Process mining: overview and outlook of Petri net discovery algorithms. In: Jensen K, van der Aalst WMP (eds) Transactions on petri nets and other models of concurrency II, vol 5460 of lecture notes in computer science, Springer, pp 225–242. 10.1007/978-3-642-00899-3_13

[28] Bottrighi A, Canensi L, Leonardi G, Montani S, Terenziani P (2018) Interactive mining and retrieval from process traces. Expert Syst App 110:62–79. 10.1016/j.eswa.2018.05.041 10.1016/j.eswa.2018.05.041

[29] Dixit PM, Verbeek HMW, Buijs JCAM, van der Aalst WMP (2018) Interactive data-driven process model construction. In: Trujillo JC, Davis KC, Du X, Li Z, Ling TW, Li G, Lee ML (eds) Conceptual modeling, vol 11157 of lecture notes in computer science, Springer International Publishing, Cham, pp 251–265. 10.1007/978-3-030-00847-5_19

[30] Beerepoot I, Di Ciccio C, Reijers HA, Rinderle-Ma S, Bandara W, Burattin A, Calvanese D, Chen T, Cohen I, Depaire B, Di Federico G, Dumas M, van Dun C, Fehrer T, Fischer DA, Gal A, Indulska M, Isahagian V, Klinkmüller C, Kratsch W, Leopold H, van Looy A, Lopez H, Lukumbuzya S, Mendling J, Meyers L, Moder L, Montali M, Muthusamy V, Reichert M, Rizk Y, Rosemann M, Röglinger M, Sadiq S, Seiger R, Slaats T, Simkus M, Someh IA, Weber B, Weber I, Weske M, Zerbato F (2023) The biggest business process management problems to solve before we die. Comput Ind 146:103837. 10.1016/j.compind.2022.103837

[31] Schuster D, van Zelst SJ, van der Aalst WMP (2023) Cortado: a dedicated process mining tool for interactive process discovery. SoftwareX 22. 10.1016/j.softx.2023.101373

[32] Schuster D, van Zelst SJ, van der Aalst WMP (2020) Incremental discovery of hierarchical process models. In: Dalpiaz F, Zdravkovic J, Loucopoulos P (eds) Research challenges in information science, vol 385 of lecture notes in business information processing, Springer, Cham, pp 417–433. 10.1007/978-3-030-50316-1_25

[33] Rojas E, Munoz-Gama J, Sepúlveda M, Capurro D (2016) Process mining in healthcare: a literature review. J Biomed Informat 61:224–236. 10.1016/j.jbi.2016.04.007 10.1016/j.jbi.2016.04.007 27109932

[34] de Roock E, Martin N (2022) Process mining in healthcare - an updated perspective on the state of the art. J Biomed Informat 127:103995. 10.1016/j.jbi.2022.103995 10.1016/j.jbi.2022.103995 35077900

[35] Dallagassa MR, dos Santos Garcia C, Scalabrin EE, Ioshii SO, Carvalho DR (2022) Opportunities and challenges for applying process mining in healthcare: a systematic mapping study. J Ambient Intell Human Comput 13(1):165–182. 10.1007/s12652-021-02894-7 10.1007/s12652-021-02894-7

[36] Xu H, Pang J, Yang X, Ma L, Mao H, Zhao D (2020) Applying clinical guidelines to conformance checking for diagnosis and treatment: a case study of ischemic stroke. In: 2020 IEEE International conference on bioinformatics and biomedicine (BIBM), IEEE, pp 2125–2130. 10.1109/BIBM49941.2020.9313532

[37] Metsker O, Kesarev S, Bolgova E, Golubev K, Karsakov A, Yakovlev A, Kovalchuk S (2019) Modelling and analysis of complex patient-treatment process using GraphMiner toolbox. In: Rodrigues JMF, Cardoso PJS, Monteiro J, Lam R, Krzhizhanovskaya VV, Lees MH, Dongarra JJ, Sloot PM (eds) Computational science – ICCS 2019, vol 11540 of lecture notes in computer science, Springer International Publishing, Cham, pp 674–680. 10.1007/978-3-030-22750-0_65

[38] Martin N, Martinez-Millana A, Valdivieso B, Fernández-Llatas C (2019) Interactive data cleaning for process mining: a case study of an outpatient clinic’s appointment system. In: Di Francescomarino C, Dijkman R, Zdun U (eds) Business process management workshops, vol 362 of lecture notes in business information processing, Springer, pp 532–544. 10.1007/978-3-030-37453-2_43

[39] de Bleser L, Depreitere R, de Waele K, Vanhaecht K, Vlayen J, Sermeus W (2006) Defining pathways. J Nursing. Manage 14(7):553–563. 10.1111/j.1365-2934.2006.00702.x 10.1111/j.1365-2934.2006.00702.x 17004966

[40] Dumas M, La Rosa M, Mendling J, Reijers HA (2018) Fundamentals of business process management, 2nd Edition, Springer, Berlin, Heidelberg. 10.1007/978-3-662-56509-4

[41] Benevento E, Dixit PM, Sani MF, Aloini D, van der Aalst WMP (2019) Evaluating the effectiveness of interactive process discovery in healthcare: a case study. In: Di Francescomarino C, Dijkman R, Zdun U (eds) Business process management workshops, vol 362 of lecture notes in business information processing, Springer, pp 508–519. 10.1007/978-3-030-37453-2_41

[42] de Weerdt J, Wynn MT (2022) Foundations of process event data. In: van der Aalst WMP, Carmona J (eds) Process mining handbook, vol 448 of lecture notes in business information processing, Springer, pp 193–211. 10.1007/978-3-031-08848-3_6

[43] van Dongen BF, BPI challenge (2012) - event log. 10.4121/uuid:3926db30-f712-4394-aebc-75976070e91f

[44] van Dongen BF, BPI challenge (2017) - event log. 10.4121/uuid:5f3067df-f10b-45da-b98b-86ae4c7a310b

[45] van der Aalst WMP, Santos L (2022) May I take your order?. In: Marrella A, Weber B (eds) Business process management workshops, vol 436 of lecture notes in business information processing, Springer, pp 99–110. 10.1007/978-3-030-94343-1_8

[46] Schuster D, Schade L, van Zelst SJ, van der Aalst WMP (2022) Visualizing trace variants from partially ordered event data. In: Munoz-Gama J, Lu X (eds) Process mining workshops, vol 433 of lecture notes in business information processing, Springer, pp 34–46. 10.1007/978-3-030-98581-3_3

[47] Schuster D, Zerbato F, van Zelst SJ, van der Aalst WM (2024) Defining and visualizing process execution variants from partially ordered event data. Inf Sci 657:119958. 10.1016/j.ins.2023.119958

[48] Chinosi M, Trombetta A (2012) BPMN: an introduction to the standard. Comput Stand Inter 34(1):124–134. 10.1016/j.csi.2011.06.002 10.1016/j.csi.2011.06.002

[49] Salimifard K, Wright M (2001) Petri net-based modelling of workflow systems: an overview. Euro J Oper Res 134(3):664–676. 10.1016/S0377-2217(00)00292-7 10.1016/S0377-2217(00)00292-7

[50] van der Aalst WMP (1998) The application of Petri nets to workflow management. J Circ Syst Comput 08(01):21–66. 10.1142/S0218126698000043 10.1142/S0218126698000043

[51] Dumas M, La Rosa M, Mendling J, Reijers HA (2013) Fundamentals of business process management. Springer, Berlin, Heidelberg. 10.1007/978-3-642-33143-5

[52] Schuster D, Martini M, van Zelst SJ, van der Aalst WMP (2022) Control-flow-based querying of process executions from partially ordered event data. In: Troya J, Medjahed B, Piattini M, Yao L, Fernández P, Ruiz-Cortés A (eds) Service-oriented computing, vol 13740 of lecture notes in computer science, Springer, Cham, pp 19–35. 10.1007/978-3-031-20984-0_2

[53] Carmona J, van Dongen BF, Solti A, Weidlich M (2018) Conformance checking. Springer. 10.1007/978-3-319-99414-7 10.1007/978-3-319-99414-7

[54] IA of Medical Oncology (2021). https://www.iss.it/documents/20126/8403839/LG%20149_Polmone_agg2021

[55] van Eck ML, Lu X, Leemans SJJ, van der Aalst WMP (2015) PM^2^: a process mining project methodology. In: Zdravkovic J, Kirikova M, Johannesson P (eds) Advanced information systems engineering, vol 9097 of lecture notes in computer science, Springer, pp 297–313. 10.1007/978-3-319-19069-3_19

[56] Accorsi R, Lebherz J (2022) A practitioner’s view on process mining adoption, event log engineering and data challenges. In: van der Aalst WMP, Carmona J (eds) Process mining handbook, vol 448 of lecture notes in business information processing, Springer, pp 212–240. 10.1007/978-3-031-08848-3_7

[57] Schuster D, van Zelst SJ, van der Aalst WMP (2021) Freezing sub-models during incremental process discovery. In: Ghose A, Horkoff J, Silva Souza VE, Parsons J, Evermann J (eds) Conceptual modeling, vol 13011 of lecture notes in computer science, Springer, pp 14–24. 10.1007/978-3-030-89022-3_2

[58] Leemans SJJ, Fahland D, van der Aalst WMP (2013) Discovering block-structured process models from event logs - a constructive approach. In: Application and theory of petri nets and concurrency, vol 7927, Springer Berlin Heidelberg, pp 311–329. 10.1007/978-3-642-38697-8_17

[59] Martin N, Fischer DA, Kerpedzhiev GD, Goel K, Leemans SJJ, Röglinger M, van der Aalst WMP, Dumas M, La Rosa M, Wynn MT (2021) Opportunities and challenges for process mining in organizations: results of a Delphi study. Bus Inf Syst Eng 63(5):511–527. 10.1007/s12599-021-00720-0 10.1007/s12599-021-00720-0

